# Hybrid de novo tandem repeat detection using short and long reads

**DOI:** 10.1186/1755-8794-8-S3-S5

**Published:** 2015-09-23

**Authors:** Guillaume Fertin, Géraldine Jean, Andreea Radulescu, Irena Rusu

**Affiliations:** 1 LINA UMR CNRS 6241 Univeristy of Nantes, Nantes, France

**Keywords:** tandem repeat, second-generation sequencing, third-generation sequencing, de Bruijn graph

## Abstract

**Background:**

As one of the most studied genome rearrangements, tandem repeats have a considerable impact on genetic backgrounds of inherited diseases. Many methods designed for tandem repeat detection on reference sequences obtain high quality results. However, in the case of a de novo context, where no reference sequence is available, tandem repeat detection remains a difficult problem. The short reads obtained with the second-generation sequencing methods are not long enough to span regions that contain long repeats. This length limitation was tackled by the long reads obtained with the third-generation sequencing platforms such as Pacific Biosciences technologies. Nevertheless, the gain on the read length came with a significant increase of the error rate. The main objective of nowadays studies on long reads is to handle the high error rate up to 16%.

**Methods:**

In this paper we present MixTaR, the first de novo method for tandem repeat detection that combines the high-quality of short reads and the large length of long reads. Our hybrid algorithm uses the set of short reads for tandem repeat pattern detection based on a de Bruijn graph. These patterns are then validated using the long reads, and the tandem repeat sequences are constructed using local greedy assemblies.

**Results:**

MixTaR is tested with both simulated and real reads from complex organisms. For a complete analysis of its robustness to errors, we use short and long reads with different error rates. The results are then analysed in terms of number of tandem repeats detected and the length of their patterns.

**Conclusions:**

Our method shows high precision and sensitivity. With low false positive rates even for highly erroneous reads, MixTaR is able to detect accurate tandem repeats with pattern lengths varying within a significant interval.

## Background

Tandem repeats are one of the most studied genome rearrangements [1-3]. Frequently located in genes or regulatory regions, tandem repeats (TR) play an important role in gene expression, genome evolution and transcriptional regulation [2-5]. They represent an important source of genome variation, help determine individuals inherited traits and are involved in genetically inherited diseases [6].

A TR is a sequence from a genome made of several (not necessarily exact) copies of the same pattern, located next to each other. In the case where each copy is identical to the pattern, the sequence is called an *exact tandem repeat *(ETR). Otherwise, it is called an *approximate tandem repeat *(ATR). Depending on the pattern length, TR are classified into microsatellites (1-10 bp), minisatellites (10-100 bp) and satellites (*>*100 bp) [7].

The problem of TR detection has been the subject of many studies due to the TR important roles. The search of TR in genomes can be done in two contexts: either in the case where a reference sequence exists or in the *de novo *context (*i.e*. without a reference sequence).

TR detection on reference sequences of genomes, chromosomes or other types of assembled DNA sequences can be performed by a significant number of existing tools (see review [8] for examples). Most of these software contain two main steps. At first, potential TR are searched with either an exhaustive [9,10] or a heuristic algorithm [11]. Some of these search methods are specifically designed for ETR such as BWtrs [9], while others can search for both ETR and ATR such as TRF [11] and mreps [10]. A filtering step is then executed for identifying the biologically significant repeats. Simple methods such as a length threshold [10] or more complex ones such as statistics-based models [9] may be used. For sake of simplicity we call all these methods *TR sequence search *tools.

In the case of a de novo context, TR detection remains a difficult problem [12]. A sequencing platform produces numerous possible overlapping sequences, called *reads*, from the initial DNA sequence (called the target DNA sequence). Developed in 2005, the second-generation sequencing (SGS for short) technologies were developed to lower the sequencing time and cost (see [13] for a description). However, the resulting reads have short length (100 bp to 250 bp) and high error rate. One of the first sequencing technology, Sanger, can have an error rate as low as 0.001%, while reads from SGS technology can have up to 2.8% errors [14]. The loss of information, due both to the short length of the reads and the error rate, is counterbalanced with high values for the *coverage depth*, that is, the average number of reads covering an arbitrary position in the target DNA sequence. In this context, obtaining a correct de novo assembly of SGS data sets from complex genomes represents a difficult challenge [15]. Indeed, the numerous repeated sequences occurring in a genome are a main cause of errors in an assembly [16-18].

In order to surpass these challenges, several de novo assemblers were developed to improve the repeat assembly (see [19-21] for examples). Their procedures are usually executed at the end of the assembly process and are especially developed for repeat assembly by using *paired-end *information [12]. Most SGS platforms propose paired-end sequencing protocols which provide pairs of reads. The reads from a pair are separated on the target DNA sequence by a known approximate distance called *insert size *(usually ranging from 500 bp to 1 kbp). The insert size being much longer than the read length, the paired-end reads are used to span regions that contain long repeats. The de novo assemblers using this type of data are able to assemble a part of the repeats of the original genome, but in their final output many repeats are still missing [12]. Our previous study [22] focuses on these missing repeats by retrieving ETR left unresolved by the assemblers. The algorithm presented in this study, called DExTaR, improves the detection of ETR after a de novo de Bruijn assembly.

The read length limitation of SGS was tackled by the Pacific Biosciences (PacBio) technologies [23]. As part of the third-generation sequencing platforms, PacBio long reads have a variable length ranging from 1 kbp to 20 kbp. This range of lengths can easily span most repeats. Unfortunately, the read length expansion came with a significant increase in the error rate. The mean error rate is of approximately 16% [24]. Therefore, most of the research work focusing on PacBio reads uses the higher quality of SGS short reads, either for correcting the PacBio reads [25-28] or for assembling in a de novo context [29,30]. The problem of detecting TR sequences by using the long PacBio reads has been analysed in the context of an existing reference sequence [31]. Also, studies show that complex regions of genomes can be reconstructed using PacBio reads when finishing genomes after a global assembly [32-34]. In the remaining of this article we will use the term *short reads *to describe SGS reads and *long reads *for the reads generated by the Pacific Bioscience technologies.

In this paper we present the first de novo method for TR detection which uses both short and long reads and performs only local assemblies. Our hybrid algorithm, called MixTaR, combines the high-quality of short reads and the large length of long reads for an efficient TR detection. By detecting both ETR and ATR, MixTaR goes further that DExTaR [22] in the TR detection. Moreover, unlike DExTaR, MixTaR does not need a previous global assembly.

MixTaR starts by building a de Bruijn graph from the short reads. The de Bruijn graph is constructed from the overlaps between fragments of reads of a certain length *k*, called *k-mers*. Due to this fragmentation of the reads, the de Bruijn graph allows a detailed analysis of complex regions of the target DNA sequence. In particular, a sufficiently long ETR appears in the de Bruijn graph as a cycle. Therefore, we analyse the cycles in the de Bruijn graph for possible TR candidates. This step can only provide potential TR patterns due to the reduced length of the short reads. Thus, we use the long reads to verify the existence of TR containing these patterns and to deduce the length of these TR. The corrected TR sequence is then obtained by a local greedy assembly of a selected set of short reads.

## Methods

In this paper, we present MixTaR, our solution to the DE NOVO HYBRID TANDEM REPEAT DETECTION problem, defined as follows.

DE NOVO HYBRID TANDEM REPEAT DETECTION

**Input: **A set *SR *of short reads and a set *LR *of long reads, both obtained from the sequencing of a DNA sequence *D*.

**Requires: **The set of TR from *D *involving the reads in *SR *and *LR *and their reverse complements.

**Figure 1:**
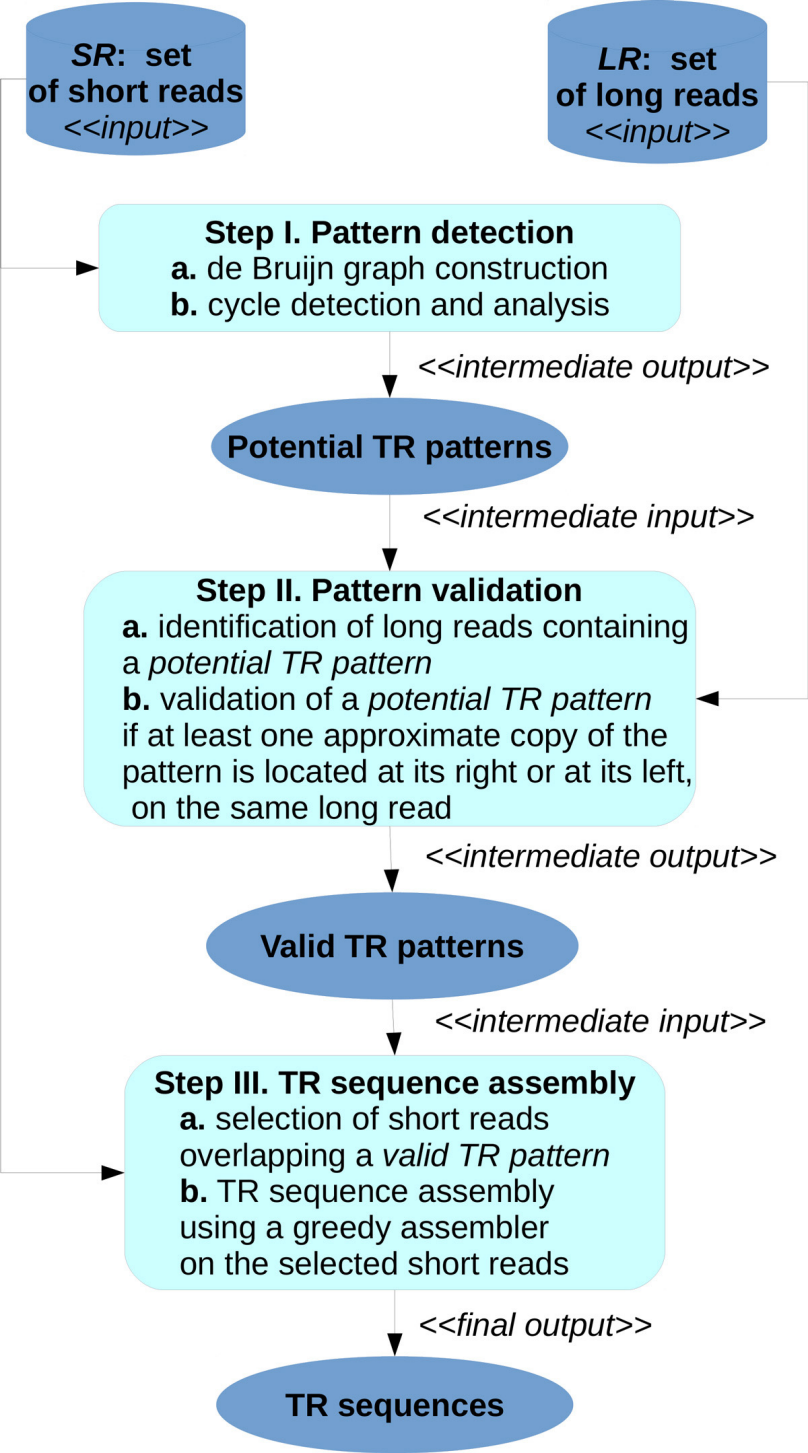
**MixTaR pipeline**.

The high-quality of the reads from *SR *and the large length of the reads from *LR *are exploited by MixTaR throughout three main steps (as presented in Figure 1): (1) pattern detection, (2) pattern validation and (3) TR sequence assembly. In the first step, we build the de Bruijn graph corresponding to the set *SR*, after including in *SR *the reverse complements of the short reads. Long repetitions, including TR, form cycles in the de Bruijn graph. Thus, we search the graph for cycle detection and by analysing the cycles we find, we deduce a list of potential TR patterns.

For the second step we add to the set *LR *the reverse complements of the long reads and we use the set to validate these potential TR patterns. Because of the significant length of the long reads, most of the TR from *D *are spanned by at least one read in *LR*. The main difficulty of using long reads is to deal with their error rate. Thus, we start by searching approximate copies of each potential TR pattern in each read from *LR*. We then validate a potential TR pattern if we identify at least two approximate copies of it located next to each other in at least one read from *LR*.

The third and final step is to identify the exact sequence of the TR containing the validated patterns. To do so, we use again the set *SR*. The short reads overlapping a validated pattern are used in a local greedy assembly, from which we obtain the precise sequence of the TR containing the pattern.

Definitions for the main notions used in the paper are given in the next paragraphs, followed by a detailed description of each step.

## Definitions

Let *s *be a string of length *|s| *over an alphabet Σ. For an integer *i *with 1 *≤ i ≤ |s|, s*[*i*] is the letter at position *i *in *s *and *s*[*i, l*] is the substring of *s *of length *l *that starts at position *i *(and thus ends at position *i *+ *l *− 1). The *prefix *and *suffix *of length *l *of the string *s *are denoted as *Pref *(*s, l) *and *Suff *(*s, l)*. For two strings *s*_1 _and *s*_2_, we denote their *concatenation *as *s*_1 _+ *s*_2_. The *alignment score *between *s*_1 _and *s*_2 _is obtained from an alignment [35] of *s*_1 _and *s*_2 _by adding 1 for each matching position, and -1 for each mismatch, insertion and deletion. The *maximum semi-global alignment score *denoted as *sgA_max _*(*s*_1_*, s*_2_) represents the highest alignment score obtained from the possible alignments of *s*_1 _and a substring of *s*_2_. A *maximum overlap alignment score oA_max_*(*s*_1_*, s*_2_*, l_min_*) between *s*_1 _and *s*_2 _is the highest alignment score obtained from the possible alignments of *Pref *(*s*_1_*, l*) and *Suff *(*s*_2_*, l*), where *l ≥ lmin*. A string *s*_1 _of length *l occurs *in a string *s*_2 _if there is a position 1 *≤ i ≤ *(*|s*_2_*| − l *+ 1) for which *s*_1 _= *s*_2_[*i, l*]. The *number of occurrences occ*(*s*_1_*, s*_2_) of a string *s*_1 _in a string *s*_2 _is the number of positions *i *for which *s*_1 _= *s*_2_[*i, l*]. In the case of a set of strings, the *number of occurrences occ*(*s, ζ*) of a string *s *in the set *ζ *of strings is equal to ∑*_t∈ζ _occ*(*s, t*).

*De Bruijn graph*. A *read *is a string over the alphabet Σ = {*A, C, G, T*}. Let *R *be a non-empty set of reads of length *l*, together with their reverse complements, obtained after sequencing a DNA sequence *D*. We denote *δ *the coverage depth used for obtaining *R*. Given a positive integer *k ≤ l*, a *k-mer *is a substring *r*[*i, k*] of a read *r ∈ R *such that 1 *≤ i ≤ l − k *+ 1. We denote *S*(*k, r*) the set of *k*-mers of a string *r*. In the case of the set *R *of reads, the set *S*(*k, R*) of *k*-mers of *R *is *∪ _r ∈ R_ S*(*k, r*). The *de Bruijn graph *[36]*G^k ^*(*R*) is the directed graph constructed from *S*(*k, R*) as follows. Each *k*-mer from *S*(*k, R*) is represented as a vertex *v *in *G^k ^*(*R*). We consider the notation *v *for both the vertex and its corresponding *k*-mer sequence. An arc *α *= (*v_i_, v_j_) *from *v_i _*to *v_j _*is built between two *k*-mers iff *Suff *(*v_i _, k − 1) *= *Pref *(*v_j _, k −1) *and *occ*(*v_i _*+*v_j _*[*k*]*, R*) *≥ *1. Because of this arc construction, each read in *R *corresponds to an oriented path in *G^k ^*(*R*). The *frequency of an arc α *= (*v_i_, v_j_*) *in G^k ^*(*R*) is *f *(*α, R*) = *occ*(*v, R*)*/δ *where *v *= *v_i _*+ *v_j _*[*k*]. Indeed, we consider that the frequency of an arc is equal to the number of times the arc has to be traversed for assembling *D*.

*Tandem Repeat*. A *pattern p *is a string of length *|p| ≥ *2 over the alphabet Σ = {*A, C, G, T*}. An *exact tandem repeat *(ETR) of the pattern *p *is a DNA sequence *ε *consisting of two or more consecutive copies of *p*, each copy being identical to *p*. Otherwise, if the copies of *p *in *ε *are not identical to each other but the maximum alignment score between each copy of *p *and *p *remains above a specific threshold, then *ε *is an *approximate tandem repeat *(ATR) of the pattern *p*. A *tandem repeat *(TR) of a pattern *p *is either an ETR or an ATR of *p*.

Let *P *= {*p*_1_, *p*_2_, . . . , $p_{n_{p}}$} be the list of copies of *p *in *ε*. When *p *has at least one complete copy in *ε*, its last copy $p_{n_{p}}$ can be partial, meaning that $p_{n_{p}}$ can be a copy (exact or approximate depending on the type of TR of *ε*) of a prefix of *p*. The *copy number cn_p _*of *p *in *ε *is the decimal number *cn_p _*= (*n_p _− *1) + |$p_{n_{p}}$|/|p| and *ε *is then defined by the pair (*p, cn_p_*). In this article, we consider that for every 1 *≤ i ≤ n*_*p*_, *p_i _*is primitive [37], meaning that *p_i _*does not contain itself an ETR. Moreover, when searching for TR in a target DNA sequence, we look for maximal TR in the sense that *cn_p _*is maximal.

**Figure 2:**
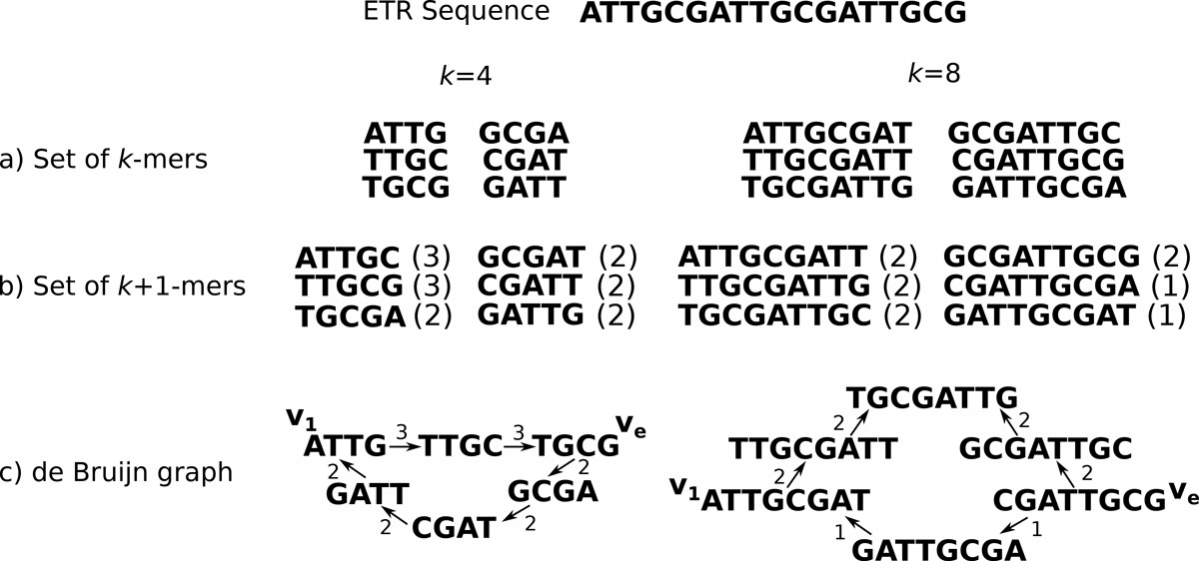
**The de Bruijn graph of an ETR with *p *= ATTGCG and *cnp *= 3 in two cases: *|p|>k *(*k *= 4, left) and *|p|≤k *(*k *= 8, right)**. The vertices *v_1 _*and *ve *are the ones described in Property 1. The arcs frequencies in the de Bruijn graph are given by the (*k*+1)-mer occurrences number in the ETR (between brackets in b)). For sake of simplicity we choose for the coverage depth *δ *= 1.

*ETR in the de Bruijn graph*. Let *k *be a positive integer and *ε *be an ETR of a pattern *p *such that *|ε| ≥ |p| *+ *k*. Let *S*(*k, ε*) = {*v_1_, v_2_, . . . , v_|p|_*} be the set of *k*-mers of *ε *such that *vi *= *ε*[*i, k*], 1 *≤ i ≤ |p|*. The set *S*(*k, ε*) is composed only of the *|p| *first *k*-mers from *ε*. Indeed, since every *|p|*-th positions in *ε *we retrieve again *p *or a prefix of *p*, after *|p| *bp in *ε *we obtain *k*-mers that are already in *S*(*k, ε*). The de Bruijn graph *G^k ^*(*ε*), built from *S*(*k, ε*), thus consists of an elementary cycle *c *(*i.e*. each node appears only once in *c*) where its nodes are linked as follows. An elementary path is constructed from *v_1 _*to *v_|p|_*since *Suff *(*vi, k − 1) *= *Pref *(*v_i_*+1, *k − 1*), for 1 *≤ i < |p|*. Because of the condition *|ε| ≥ |p| *+ *k*, we deduce that *ε*[*p *+ 1*, k*] = *p*[1*, k*] = *ε*[1*, k*] and thus *Suff *(*v_|p|_, k − 1) *= *Pref *(*v_1_, k − 1*). Therefore, *v_|p|_*is also connected to *v*_1 _by the arc (*v_|p|_, v_1_*). An example is presented in Figure 2. Moreover, for every such cycle, Property 1 below is satisfied (see also Figure 2).

**Property 1 (Arc frequency property) ***Let v_1 _*= *ε*[1*, k*] *and ve *= *Suf f *(*ε, k*) *be the first and last k-mers of the ETR ε *= (*p, cnp*) *with |ε| ≥ |p| *+ *k. In the cycle formed by ε in G^k ^*(*ε*)*, the frequency of each arc of the path from v_1 _to v_e _is the same. If × denotes this frequency, then the frequency of each arc of the path from v_e _to v_1 _is equal to × − *1.

Property 1 is respected for both *|p| ≤ k *and *|p| > k *as illustrated in Figure 2 for two different values of *k*. In both cases, the number of vertices in the cycle is equal to *|p| *due to the unique presence of each *k*-mer in a de Bruijn graph.

Now that the definitions used in this paper are presented, we describe in detail the three main steps of MixTaR pipeline (Figure 1).

## Pattern detection

*SR *is the set of short reads together with their reverse complements obtained after sequencing the DNA sequence *D*. As mentioned before, the ETR from *D *with length at least *|p| *+ *k *(where *p *is the pattern of the ETR) form in a de Bruijn graph *G^k ^*(*SR*) elementary cycles respecting Property 1. These ETR can represent substrings of longer ATR of the same pattern *p *in *D*. In this case, approximate copies of *p *are located next to the ETR in *D *and the ETR is considered as *internal *to a longer TR. In the following, these TR that contain an internal ETR forming a cycle in the de Bruijn graph are named *robust TR*. Our algorithm MixTaR first searches for ETR, then for the robust TR containing them.

*Cycle search algorithm *We consider that each elementary cycle in de Bruijn graph represents a potential ETR from *D*. Thus, after constructing the de Bruijn graph *G^k ^*(*SR*) for a specific value of *k*, we start searching for elementary cycles in *G^k ^*(*SR*). The sequencing errors in the set *SR *may introduce erroneous vertices in *G^k ^*(*SR*). In order to eliminate them, we consider in our search only the vertices for which *occ*(*v, SR*) *≥ σ*, where *σ *is a parameter with a value depending on *δ*, the coverage depth of *SR*. Moreover, we sort the list of vertices in *G^k ^*(*SR*) in a descending order of their number of occurrences in *SR*.

A de Bruijn graph of a complex organism has a significant size, with hundreds of thousands of cycles. Hence, in order to detect a maximum number of elementary cycles in a limited amount of time, we use one of the most efficient cycle detection methods, namely Johnson's algorithm [38]. Johnson's algorithm explores the graph from every vertex *v *and returns the cycles that contain *v *and that are not yet detected. To limit this exploration we introduce three parameters: *η*, Λ*_max _*and *λ_max_*. From each vertex *v *we start by searching the cycles of maximal length of Λ*_max_*. After exploring *η *arcs from *v *and if there are still arcs to be explored, the algorithm searches for the remaining cycles from *v *of maximal length of *λ_max _*(*λ_max _≤ *Λ*_max_*).

As mentioned before and explained in the next paragraph, after analysing a cycle *c *of *l *vertices we can obtain a pattern *p *of length 2 *≤ |p| ≤ l*. Therefore, in order to obtain patterns with significant lengths, we have to maximize the number of cycles potentially containing an ETR of maximal length of Λ*_max_*. For this, we consider that the arcs of the cycles containing an ETR have a high value for their frequency in *SR*. Thus, from each vertex we start by exploring its output arcs in descending order of their frequency in *SR*. In this way, we traverse the arcs having the highest frequency in the first part of our cycle search. The parameters Λ*_max_, η *and *λ_max _*are set depending on the complexity of *G^k ^*(*SR*) and on the running time allowed by the user for the cycle search.

Each vertex *v *from which we start the exploration of the graph has to satisfy an additional condition: *occ*(*v, LR*) *>*0. Because of the order in which vertices are considered, the vertex *v *from which the algorithm searches from cycles is also the vertex with the highest number of occurrences in *SR *from the cycles obtained for *v*. We then consider that we have for *v *the highest chance to find an errorless occurrence in *LR *between the vertices of the detected cycles from *v*. This additional condition is used in the second step of MixTaR, to validate the obtained patterns using the set *LR*.

*Cycle analysis*. Each cycle detected by Johnson's algorithm is analysed in order to find a potential ETR. The difficulty is that in a de Bruijn graph *G^k ^*(*SR*), a cycle is formed by a repeat, but not necessarily by an ETR. For identifying the cycles formed by an ETR, we determine the ones that respect Property 1. Let *c *be a cycle in *G^k ^*(*SR*) such that *c *is formed by an ETR *ε *from the target DNA fragment *D*. We consider *v_1 _*= *ε*[1*, k*] and *v_e _*= *Suf f *(*ε, k*) to be the first and last *k*-mer of *ε *as presented in Property 1. The flanking regions of *ε *in *D *create two arcs *α_in_*= (*x, v_1_*) and *α_out _*= (*v_e_, y*) is *G^k ^*(*SR*), such that *x*[1] + *ε *+ *y*[*k*] occurs in *D*. We consider that these two arcs mark the beginning and the end of *ε*.

In the case of a complex *D, ε *or substrings of *ε *can appear several times at different locations in *D*. In the following we call these sequences *additional interspersed repeats *(AIR) of *ε*. In a de Bruijn graph, each vertex represents a unique *k*-mer. Since the AIR and *ε *have common *k*-mers, their corresponding paths in *G^k ^*(*SR*) share common vertices and thus parts of *c*. This fact has two consequences for *c*. The first one is that, as is the case for *ε*, the flanking regions of the AIR can create additional input and output arcs from the vertices of *c*. Since the AIR occur in *D*, they are spanned by reads in *SR*. Thus, the second consequence is that the arcs of the ETR in common with an AIR have a frequency corresponding to both the ETR and the AIR. Therefore, in order to detect if a cycle from *G^k ^*(*SR*) contains an ETR we have to remove the impact of the potential AIR from the frequencies of the cycle arcs. The method, described in Algorithm 1 detailed below, is applied for each detected cycle *c*.

**Algorithm 1: **Cycle frequency cleaning

**Input**: The sets *Vc *and *Ac *of vertices and arcs of a cycle *c*; the sets *A_in_ − *{*α_in_*} and *A_out _− *{*α_out_*} of input and output arcs of the vertices in *c*

**Output**: The frequency of the arcs in *c *corresponding only to the ETR marked by (*α_in_, α_out_*);

1 *f_acc_ ← *0;

2 **for ***i ← *1 **to ***|Ac| ***do**

3     **if ***i *= *|Ac| ***then**

4     *αi ← *(*vi, v_1_*)*, αi ∈ Ac*;

5     **else**

6     *αi ← *(*vi, vi*+1)*, αi ∈ Ac*;

7     *f_arc_*[*αi*] *← f *(*αi, SR*);

8     *f_input_*[*vi*] *← *$\underset{\begin{array}{c} q=\left(x,v_{i}\right)\\ q\in A_{out}-|\left\{\alpha_{in}\right\} \end{array}}{\sum }f\left(q,SR\right)$

9     *f_output_*[*v_i_*] *← *$\underset{\begin{array}{c} u=\left(v_{i},y\right)\\ u\in A_{out}-\left\{\alpha_{out}\right\} \end{array}}{\sum }f\left(u,\;SR\right)$

10     *f_acc _← max*(*f_acc _*+ *f_input_*[*vi*] *− f_output_*[*vi*], 0);

11     *f_arc_*[*α_i_*] *← f_arc_*[*α_i_*] *− f_acc_*;

12     *f_output_*[*v_i_*] *← max*(*−f_acc_*, 0);

13     *f_input_*[*v_i_*] *← *0;

14 **if ***f_acc _>*0 **then**

15     **for ***i ← *1 **to ***|Ac| ***do**

16         **if ***i *= *|A_c_| ***then**

17         *α_i _← *(*v_i_, v_1_*)*, αi ∈ A_c_*;

18         **else**

19         *α_i _← *(*v_i_, v_i_*+_1_)*, α_i _∈ A_c_*;

20         *f_acc _← max*(*f_acc _− f_output_*[*vi*], 0);

21         *f_arc_*[*α_i_*] *← f_arc_*[*α_i_*] *− f_acc_*;

22 return *f_arc_* [];

Let *V_c _*be the set of vertices of *c *and *A_c _*be the set of its arcs. We start by computing for each cycle *c *the set of input arcs *A_in _*= {(*x, v*) with *x ∉ V_c _*and *v ∈ Vc*} and the set of output arcs *A_out _*= {(*v, y*) with *v ∈ Vc *and *y ∉ V_c_*}. Unfortunately, the paths of the ETR and the AIR are not known. Thus, we suppose that each couple (*α_in_, α_out_*) *∈ A_in_*x*A_out _*is a potential marker for the beginning and the end of an ETR *ε *in *D*, corresponding to the cycle *c*. Therefore, the arcs in *A_in _− *{*α_in_*} *∪ A_out _− *{*α_out_*} are the endpoints of the paths defined by all the AIR of *ε*.

In the beginning, the frequency *f_arc_*[*α*] of an arc *α ∈ Ac *is initialized to *f *(*α, SR*) (computed from the number of occurrences of the corresponding (*k*+1)-mer in *SR *as described before). We start traversing *c *and for each vertex *v_i _∈ V_c_*, we compute the incoming frequency *f_input_*[*v_i_*] and the outgoing frequency *f_output_*[*v_i_*]. We then use a cumulative frequency *f_acc _*to remove the impact of the AIR from the arcs in *c*. Initially, the cumulative frequency *f_acc _*is equal to 0. For each vertex *v_i _*we add to *f_acc _*the difference between *f_input_*[*v_i_*] and *f_output_*[*v_i_*]. If *f_acc _>*0 then its value represents the frequency of the AIR, and thus in excess, of the arc *α_i _*= (*v_i_*, *v*_*i*+1_) and we remove it from *f_arc_*[*α_i_*]. Otherwise, its absolute value corresponds to the frequency in excess of the arc *α_i _*= (*v*_*i−*1_, *v*_*i*_) and it is removed in a second pass through the cycle. In this case, *f_output_*[*v_i_*] = *|f_acc_| *and we set *f_acc _*= 0. In both cases, we set *f_input_*[*v_i_*] = 0 since its impact is now included in *f_acc_*.

In order to deduce a potential ETR pattern from *c*, the remaining frequency of the arcs of *c *has to respect Property 1. In this case, we construct the ETR *ε *of the pattern *p *spelled by the cycle *c *in the following manner. Let *l *be the number of vertices of *c*, then *ε *= *v_1 _*+ *v*_2_[*k*] + . . . + *v*_*l*_[*k*]. As mentioned before, *l *= *|p| *and thus *p *= *ε*[1*, l*].

### Pattern validation

The cycle analysis we just described can only return potential patterns of ETR, and thus, of robust TR. However, some of the information given by the short reads is lost because of their hashing into *k*-mers. As a consequence, the set of patterns obtained from the de Bruijn graph may contain false positives. Hence, for each obtained pattern we have to validate its presence in at least one TR from the target DNA fragment *D*. For this, we use the set *LR *because of the significant length of its reads.

With a variable length that can range from 1 kbp to 20 kbp [23], the reads in *LR *can span most TR in *D*. The hypothesis that we make is weaker: we assume that each pattern *p *of a TR has at least two neighbouring copies spanned by at least one read in *LR*. Thus, we validate a pattern if we identify at least two copies of it located next to each other in at least one long read. Otherwise the pattern is considered as a false positive and is discarded.

The difficulty raised by this approach is to correctly identify two neighbouring copies of a pattern in spite of the high error rate of long reads, which is of approximately 16%. A possible solution is the use of a long read correction procedure [25-28]. However, in case of complex genomes, even the most efficient methods are not able to correct all the errors from the long reads (see Results and discussion section for more details). Thus, our pattern search method allows the use of corrected and also non-corrected long reads.

In order to validate a pattern *p*, we start by searching an approximate copy of *p *in the reads in *LR*. Let *c *be a cycle detected at the previous step, and let *v *be the vertex of *c *having the highest number of occ in *SR*. As described in the previous step, *v *satisfies the condition *occ*(*v, LR*) *>*0. Let *p *be the potential pattern deduced from the analysis of *c, i.e. p *is the pattern of the ETR spelled by *c*. If *|p| < k *or if *v *= *Suff *(*p, x*) + *Pref *(*p, k − x*) for an integer 1 *≤ × < k*, then *v *occurs in a string obtained by successive concatenations of *p*. Otherwise, *v *occurs directly in *p*. For a fast validation of the pattern *p*, we limit the search for copies of *p *to each long read *r *containing *v*. Let *s *be a string initially identical to *p*. If *occ*(*v, s*) = 0, we extend *s *by successive concatenations of *p *in such way that *s *is the shortest string for which *occ*(*v, s*) *>*0. The process is finite since *v *occurs in the ETR of *p *spelled by *c*.

Let *pos *be a position in a long read *r *such that *r*[*pos, k*] = *v*. We then try to identify an occurrence of *s *in *r *by searching for the alignment with the highest score between *s *and a substring of *r *around *pos*. Approximately 70% of the errors in *LR *are insertions [24]. Thus, we consider for our alignment a substring of length 2*|s| *from *r *and we compute the maximum semi-global alignment score *t *= *sgA_max_*(*s, r*[*pos − |s|*, 2*|s|*]). We then use a parameter *τ *, which is a threshold for our score *t*. The value of *τ *is set according to the approximate percentage of errors of the long reads we used, raw or corrected. If *t/|s| < τ *, then *p *is considered as a false positive. Otherwise, either *occ*(*p *+ *p, s*) *>*0 and the pattern *p *is validated or we repeat the process for *s *= *s *+ *p*.

### TR sequence assembly

Because of the high error rate of long reads, the partial TR detected in the second step of MixTaR contains a significant amount of erroneous bases. Thus, in the last step of our algorithm, we need to find the exact sequence for each TR. For this, we use once again the set *SR*. The TR of each pattern *p *are constructed by a local assembly of the set *SR_p _*of short reads overlapping *p*.

To construct the set *SR_p _*for each *p *we introduce two parameters *γ *and *ϑ*.

The smaller the length of the pattern *p *is, the higher is the probability that a read in *SR *overlaps *p*. Hence, the size of *SR_p _*and the running time of its assembly can be significant for short *p*. Let *s *be a string initially identical to *p*. In order to limit the number of short reads *r *used in the local assemblies, if *|s| < γ*, we extend *s *by successive concatenations of *p *such that *s *is the shortest string for which *|s| ≥ γ*. The value for *γ *depends on the length of the short reads in *SR*, and has to be small enough to maximize the number of reads spanning a part of a TR of *p *included in *SR_p _*while limiting its size.

We then construct the set *SRp *by searching the short reads overlapping *s*. MixTaR allows approximate overlaps since *p *can be a pattern of an ATR. For each short read *r ∈ SR*, we compute the overlap alignment score *t *= *max*(*oA_max_*(*s, r, γ*)*, oA_max_*(*r, s, γ*)). If *t ≥ ϑ*, then *r *is added to *SR_p_*. The value for *ϑ *depends on the error rate of the reads in *SR *and on the difference allowed between a pattern and its copies for an ATR.

Once the set *SRp *is computed, we assembly it. For this, we use a greedy assembler. This choice was made because of its short running time needed in order to obtain satisfying results [15]. A greedy assembler computes a specific scoring function for the overlap between each pair of short reads. Then the short reads are assembled in larger sequences, called contigs, by successive assemblies of the short reads with the overlap with the highest score.

In each contig output by the greedy assembler, we may find several TR, and we have to identify their positions in the contig. To do so, we use a TR sequence search tool (see [8] for examples) which gives us the positions of the TR in each contig. The set of contigs is then analysed. In some cases, we can obtain contigs that do not contain any TR. Since these contigs are not significant for our TR detection, they are removed. In the case where a TR occurs at an end of a contig, we can suppose that this TR is not complete. Thus the contig becomes a *seed*. Each seed is then extended in order to obtain the complete sequence of the TR. The extension of the seeds is done using the greedy assembler with the set of reads $SR-{U_{p}}_{\in ℑ}SRp$, where  ℑ is the set of all patterns validated during the second step of MixTaR. Once all the seeds are extended, we run the TR sequence search tool for a complete identification of the TR on the set of seeds. In the end, we output the TR obtained on the seeds along with the TR from the contigs.

## Results and discussion

### Experimental setup

In this section, we present the adaptations included to MixTaR for the real case of non-homogeneous coverage depth data along with the tools and libraries used for the experiments.

For the first step of MixTaR (Step I. in Figure 1) we use the de Bruijn graph library GATB-lib [39] for the construction of the de Bruijn graph. In this step, the result of the arc frequencies computations presented in Algorithm 1 depends on the homogeneity of the coverage depth used for obtaining the set *SR*. Let *c *be a cycle in *G^k ^*(*SR*) formed by an ETR *ε *from the target DNA fragment *D*. In the real case of non-homogeneous coverage depth of *SR*, the bases of *ε *are not always covered by the same number of short reads. Thus, the frequencies of the arcs in *c *may fluctuate. Therefore, Property 1 is verified using an interval rather than a specific value for the frequency of an arc. This interval is computed based on the coverage depth for the set *SR *and on the mean frequency of the arcs in *c*.

In the pattern validation step of MixTaR (Step II. in Figure 1), we compute the maximum semi-global alignment score between the patterns and the long reads using the overlap alignment method proposed by the library SeqAn [40]. This method is also used at the end of our algorithm (Step III. in Figure 1) for computing the overlap between the patterns and the short reads. To compute the local greedy assemblies, we chose SSAKE [41] due to its low running time and simple setup needed to obtain satisfying results [17].

For our experiments we run the TR sequence search tool called mreps [10] in order to identify the TR from the contigs obtained with SSAKE. Mreps is a software tool designed for a fast identification in DNA sequences of both ETR and ATR with primitive patterns. We also used mreps for identifying TR in the reference DNA sequences of the tested organisms. Due to the significant number of TR in the tested organisms, mreps was parameterised each time to find TR with at least two complete copies.

Since the DNA strand of the contigs obtained with SSAKE, and thus of the TR detected, is not known in advance, when we detect a TR and its reverse complement, we consider that they represent the same TR. Thus, a detected TR can be represented either by its sequence or by both its sequence and its reverse complement. In our analysis we consider for the target DNA fragment *D *only its reference strand. Thus a TR has been correctly identified if we detected its sequence (alone or together with its reverse complement). Thus, each TR can be classified in:

• True Positive (*T P*) if we detect the complete sequence (*i.e*. with the right copy number) of the TR from *D*;

• True Positive Incomplete (*T P_i_*) if we detect only a part of the TR from *D*;

• False Negative (*F N) *if we do not detect the TR from *D*;

• False Positive (*F P) *if we detect the TR but its sequence does not occur in *D*.

The accuracy of our method is then measured using the two following statistics:

• *P_recision _*= (*T P *+ *T P_i_*)*/*(*T P *+ *T P_i _*+ *F P) *which measures the fraction of retrieved TR that are present in *D*;

• *Sensitivity *= (*T P *+ *TP_i_*)*/*(*T P *+ *T P_i _*+ *F N) *which measures the fraction of TR from *D *that are correctly identified.

For each step of MixTaR, the main parameters that need to be set are:

• Step 1: Pattern detection

- the length *k *of the *k*-mers for the de Bruijn graph construction.

- the minimum number of occurrences *σ *of a *k*-mer in *SR*. The value of this parameter depends on the coverage depth of *SR*.

- the maximal length Λ*_max _*of cycles detected from each vertex *v*.

- the maximal number of arcs *η *explored from each vertex *v *when looking for cycles of maximal length Λ*_max_*. The values of Λ*_max _*and *η *depend on the size of the de Bruijn graph and on the running time allowed by the user.

- the maximal length *λ_max _*of cycles detected for a vertex *v *once the maximal number of arcs *η *was visited for *v*. The value of *λ_max _*(*λ_max _≤ *Λ*_max_*) also depends on the size of the de Bruijn graph and on the running time allowed by the user.

• Step 2: Pattern validation

- the minimum alignment score *τ *allowed between a pattern and a long read. The value for this parameter depends on the error rate of the long reads.

• Step 3: TR sequence assembly

- the minimum length *γ *of the pattern used for selecting the short reads for the local assemblies. From our experiments, the best results for a length of short reads of 100 bp were obtained for *γ *= 10.

- the minimum alignment score *ϑ *allowed between a pattern and a short read and also between the pattern and its copies. From our experiments, the best results were obtained for *ϑ *= 10.

**Figure 3:**
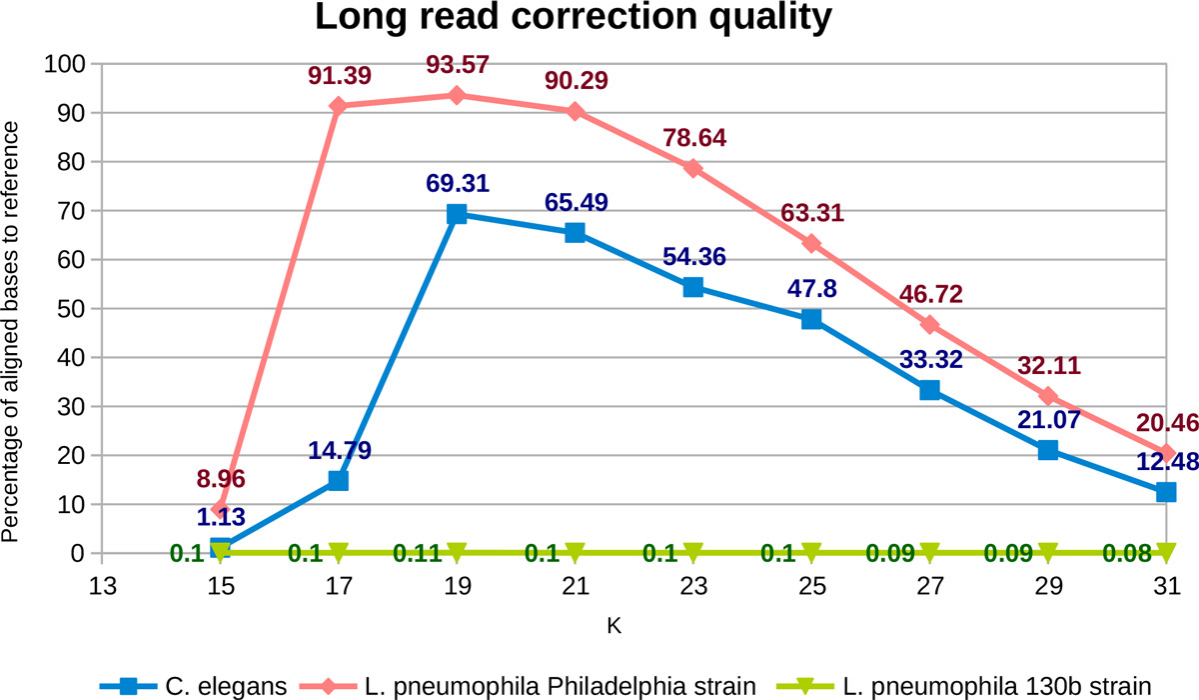
**Percentage of aligned bases of the corrected long reads on the reference DNA sequences**.

*Long read correction quality*. MixTaR is conceived in order to work with both corrected and non-corrected long reads in the input. For our experiments we used long reads that were simulated with PBSIM [42] (on the first chromosome of *C. elegans *and on the *Philadelphia *strain of *L. pneumophila*) or obtained with the PacBio sequencing technologies (for the *130b *strain of *L. pneumophila*). For a complete analysis of the impact of long reads errors on MixTaR, we corrected these sets of long reads with LoRDEC [26]. LoRDEC is a long read error correction tool based on a de Bruijn graph constructed from a short read set. The main parameter for LoRDEC is *k*, the *k*-mer length for the de Bruijn graph. We run LoRDEC with different odd values for *k ∈ *[15, 31] and aligned the corrected long reads on their reference DNA sequences with the method used by GAGE [16]. The percentage of aligned bases of the corrected long reads on their reference DNA sequences are presented in Figure 3. Each time the best results were obtained with *k *= 19, as for the results presented in the paper describing LoRDEC [26]. Even for the optimal value of *k *we can notice that, for the first chromosome of *C. elegans*, GAGE was not able to align approximately 30% of corrected long reads bases on the reference sequence [GenBank:GCA_000002985.3]. The values presented in Figure 3 are obtained for long reads with a coverage depth of 20x. For long reads with a coverage depth of 100x we obtained similar values. This high percentage of unaligned bases is due to the complex structure of the chromosome whose length is approximately 15 Mb. Mreps identifies on it a significant number of TR as will be shown in the following paragraphs. In the case of the *Philadelphia *strain of *L. pneumophila *the percentage of aligned bases is significantly higher. Because of a smaller set of repeats and the short length (3.4 Mb) of the genome [GenBank: GCA_000008485.1], the correction of the long reads is more accurate. As for the first chromosome of *C. elegans*, similar results were obtained for long reads with a coverage depth of 20x and 100x. In Figure 3 we present the ones obtained for the set with a coverage depth of 20x. For the *130b *strand of *L. pneumophila*, we corrected a set of real long reads [SRA:ERX620205]. The corrected long reads were then aligned on the draft genome constituted of 159 contigs [GenBank:GCA_000211115.2]. The low percentage of aligned bases of the corrected long reads on the draft genome is probably caused by the error percentage in the set of real long read which is higher than the announced 16% [24].

In the following, we present the results obtained with MixTaR on both simulated and real data sets. As mentioned before, our algorithm is set to analyse all the cycles of maximum length *λmax *from the de Bruijn graph and only a part of the cycles of length between *λmax *and Λ*max*. Also, the length of a pattern deduced from a cycle is equal to the number of vertices of the cycle. Hence, for the TR detected with MixTaR we present two separate analyses. At first, we describe the results obtained on the TR with a pattern length of maximum *λmax *bp. Then we extend our analysis to the TR with a pattern length of maximum Λ*max *bp. These analyses are conducted on both the set of robust TR (those that the algorithm targets) and on the set of general TR from the chromosome. Some of the general TR do not have an internal ETR that forms a cycle in the de Bruijn graph and are therefore not directly targeted but are found by MixTaR.

### Simulated data sets

For our experiments, we needed a complex organism presenting numerous TR. We chose *Caenorhabditis elegans*, which is a transparent nematode widely used as a model organism. As presented in [17], *C. elegans *is an organism for which assemblers need high computation time for a low percentage of correctly mapped contigs. This is mainly due to the important number of repeats, such as TR. Its genome was downloaded from GenBank [GenBank:GCA 000002985.3]. For our experiments, we used the first chromosome of *C. elegans*. This chromosome, of length of approximately 15 Mb, has a very complex structure and contains more TR than many complete genomes. We run mreps [10] to identify the TR from its reference sequence and we obtained a set of 39,006 TR with pattern *p *satisfying 2 *≤ |p| ≤ *100.

Paired-end short reads datasets with length of 100 bp and a coverage depth of 20x were obtained using the GemSIM read simulator [43]. To test the impact of the sequencing errors of the short reads on the results quality of MixTaR, the first dataset is errorless (*SR-NE*, for Short Reads with No Error), while the second one (*SR-E*, for Short Reads with Errors) simulates Illumina errors. Two other sets of paired-end short reads were obtained by correcting the set *SR-E*: one (*SR-CE1*, for Short Reads with Corrected Errors 1) by using the trimming tool proposed by SSAKE [41] and the other one (*SR-CE2 *for Short Reads with Corrected Errors 2) by using the correcting tool proposed by ALLPATHS [44], one of the most efficient error correction methods for short reads [16].

Long reads datasets of coverage depth of 20x and 100x were obtained using the PacBio reads simulator PBSIM [42] (*LR-E *x20 and x100, for Long Reads with Errors). Two other sets were obtained by correcting *LR-E *x20 and x100 with LoRDEC (*LR-CE *x20 and x100 Lond Reads with Corrected Errors) as presented before.

For the experiments on the first chromosome of *C. elegans*, we used the following values for the MixTaR parameters. Since the short reads have a length of 100 bp, for the de Bruijn construction we tested all the odd values for *k ∈ *[17, 47]. Due to the coverage depth of short reads (x20) we applied the cycle search algorithm for all *k*-mers with a frequency of at least *σ *= 10. The de Bruijn graph for the first chromosome of *C. elegans *has a significant number of cycles, thus for the cycle search we set *η *= 10, 000 arcs for cycles of maximal length Λ*max *= 100 and then *λmax *= 20. For the minimum alignment score *τ *we used the value *τ *= 10 for the non-corrected long reads and *τ *= 20 for the corrected ones.

In the following paragraphs, the results obtained with MixTaR are analysed from two points of view: the percentage and the pattern length range of detected TR from the first chromosome of *C. elegans*.

**Figure 4:**
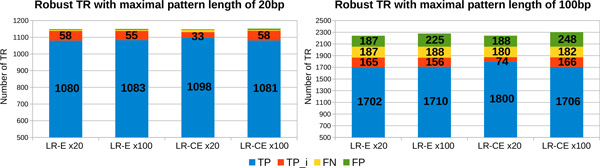
**Number of robust TR detected from the first chromosome of *C. elegans *using long reads with different error rates**. The results are obtained with the 4 sets of simulated long reads with coverage depth of x20 and x100, non-corrected *LR-E *and corrected *LR-CE*. For each run, we used the set *SR-NE *of short reads and *k *= 17.

*Percentage of detected TR depending on the quality of long reads *In order to evaluate the impact of the errors from the long reads on the quality of the results obtained by MixTaR, we run it on the four sets of long reads described in the previous paragraph, *i.e. LR-E *and *LR-CE *with a coverage depth of x20 and x100. In order to evaluate only the impact of long reads errors, we place ourself in the case of optimal conditions for the short reads. Hence, for these runs, we used the set without errors for the short reads, *SR-NE*, and different odd values for *k ∈ *[17, 47]. In the following, we present the results obtained for *k *= 17, the value for which MixTaR returns the largest set of TR. Figure 4 describes the results obtained on the set of robust TR. After running mreps on the first chromosome of *C. elegans*, we extracted, from the set of TR returned by mreps, the robust TR. For *k *= 17, we obtained 1,144 robust TR with *|p| ≤ *20 and 2,054 robust TR with *|p| ≤ *100. Each bar from Figure 4 presents the number of TR for the four types of TR presented above (*TP , TPi, FP *and *FN)*. MixTaR is able to detect the complete sequence of more than 94% of the robust TR with *|p| ≤ *20 from the chromosome. The percentage drops to 82.8% for the robust TR with *|p| ≤ *100, due to the fact that MixTaR can not explore all the cycles with length between 20 and 100 vertices. This percentage could be increased by allowing a longer running time and a higher value for the parameter *η *(*i.e*. the maximum number of arcs explored for a vertex in order to find cycles of maximal length of 100 vertices). Moreover, the percentage of FP is about 0.3% for TR with *|p| ≤ *20 and at most 12% for TR with *|p| ≤ *100. The FP obtained are due to the greedy assemblies. Even if the set of short reads is errorless, SSAKE can make wrong choices when assembling the set of reads for a TR pattern. This happens when, due to long repeats, two reads have the longest overlap but are not located in the same region in the chromosome. However, by selecting the adequate short reads for the local greedy assemblies, we are able to limit the number of FP obtained, contrary to a global assembly.

The exact values for the precision and the sensitivity obtained on the set of robust TR with *|p| ≤ *100 are presented in the first two columns of Table 1. There is no notable difference between the set of TR obtained on the four sets of long reads. This is probably due to the fact that, in the case of a complex organism with significant number of repeats, correcting long reads remains a difficult task. By using the long reads only for the validation of the TR patterns, MixTaR is robust with respect to the error rate of long reads.

**Table 1:** Precision and sensitivity values for the detection of robust TR and of general TR with maximal pattern length of 100 bp from the first chromosome of *C*.

	Robust TR		General TR	
	**Precision**	**Sensitivity**	**Precision**	**Sensitivity**

**LR-E x20**	**0.909**	0.909	0.990	**0.984**

**LR-E x100**	0.892	0.908	0.988	**0.984**

**LR-CE x20**	0.909	**0.912**	**0.993**	**0.984**

**LR-CE x100**	0.883	0.911	0.987	**0.984**

*elegans*. The results are obtained with the set *SR-NE *of short reads and *k *= 17.

**Figure 5:**
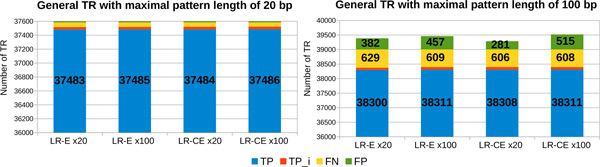
**Number of general TR detected from the first chromosome of *C. elegans *using long reads with different error rates**. The results are obtained with the 4 sets of simulated long reads with coverage depth of x20 and x100, non-corrected *LR-E *and corrected *LR-CE*. For each run, we used the set *SR-NE *of short reads and *k *= 17.

Besides the robust TR, MixTaR also detects general TR as presented in Figure 5. A main reason for the detection of general TR is the fact that many TR from the first chromosome of *C. elegans *have their pattern highly similar to at least one pattern of a robust TR. In this case, by selecting all the short reads overlapping a pattern of a robust TR, we are also able to assembly the general TR of this pattern or of a similar pattern. Another reason is the location of the general TR. Indeed, by assembling the sequences of the robust TR we obtain also their flanking regions; thus MixTaR can identify the general TR that are in these regions.

The results of mreps on the first chromosome of *C. elegans *showed 37,584 TR with *|p| ≤ *20 and 39,006 TR with *|p| ≤ *100. The results of MixTaR are even better on these sets of general TR, with more than 98% of TR detected and less than 1.3% of FP. This is due to the fact that the first chromosome of *C. elegans *contains a significant number of TR with similar patterns. By selecting the most significant short reads for the local assemblies, we are able to detect a high percentage of general TR without introducing errors. The precision and sensitivity values on the set of general TR with *|p| ≤ *100 are given on the the third and fourth column of Table 1. Once again, we observe, overall, that the correction of the long reads does not have a true influence on the quality of the results. We can notice however than the number of FP obtained with the set *LR-CE *x20 on the TR with maximal pattern length of 100 bp is at most 45% lower that the one obtained with the other three sets (see Figure 5). But this represents only about 0.6% of all the TR detected by MixTaR. Since in general the cost of correcting the reads in terms of running time and memory usage is significant, in the following we present the results obtained using the set of long reads non-corrected *LR-E *with the lowest coverage depth, x20. The differences observed between the results obtained with this set of long reads and with the three others are similar to the ones presented in this paragraph.

**Figure 6:**
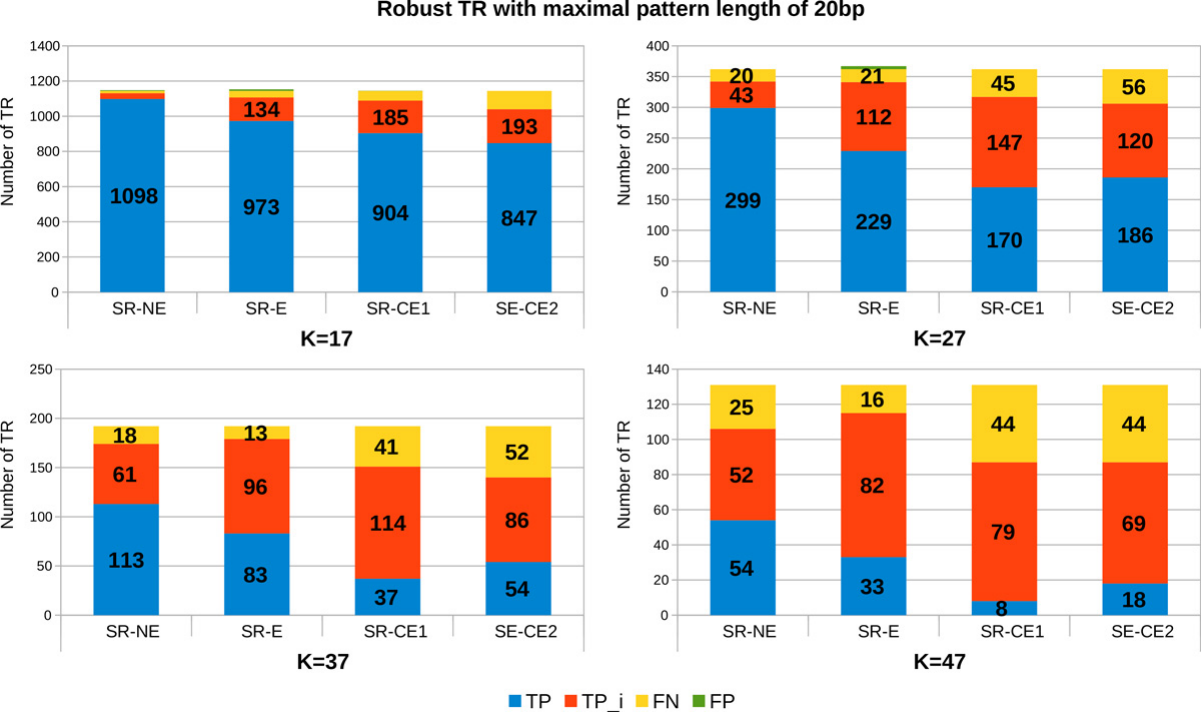
**Number of robust TR with maximal pattern length of 20 bp detected from the first chromosome of *C. elegans***. The results are obtained with the set *LR-E *of long reads with a coverage depth of x20.

*Percentage of detected TR depending on the quality of short reads and on the k-value variance*. The robustness of MixTaR with respect to the error rate in the short reads is analysed by running the algorithm on the four sets of short reads described in the previous paragraph: *SR-NE, SR-E *and the two sets of corrected reads, *SR-CE1 *and *SR-CE2*. The set of long reads used is *LR-E *with a coverage depth of x20. For each set of short reads, we executed several runs of MixTaR with different odd values of *k ∈ *[17, 47]. In this paragraph, we present the results obtained for four significant values of *k*, namely 17, 27, 37 and 47. As for the previous paragraph, we start our analysis with the set of robust TR. Figure 6 presents the results obtained for the robust TR and for which *|p| ≤ *20. The number of robust TR differs depending on the value of *k *we use. After running mreps on the first chromosome of *C. elegans*, we found 1,114 robust TR for *k *= 17, 362 robust TR for *k *= 27, 192 robust TR for *k *= 37 and the number is reduced to 131 robust TR for *k *= 47. Each bar in Figure 6 represents the set of robust TR for the respective value of *k *along with the set of FP returned by MixTaR. The best results are obtained for *k *= 17, which can be explained by the fact that the smaller the value for *k*, the higher the fragmentation of the reads. Hence, the number of TR forming a cycle in the de Bruijn graph is increasing, and our algorithm has then the possibility of analysing and detecting them. Also, the high fragmentation of the short reads obtained with small values for *k *allows a better identification of the the arc frequency variation caused by the coverage depth. Thus MixTaR is able to better identify the case when a TR pattern can be deduced from the cycle.

**Figure 7:**
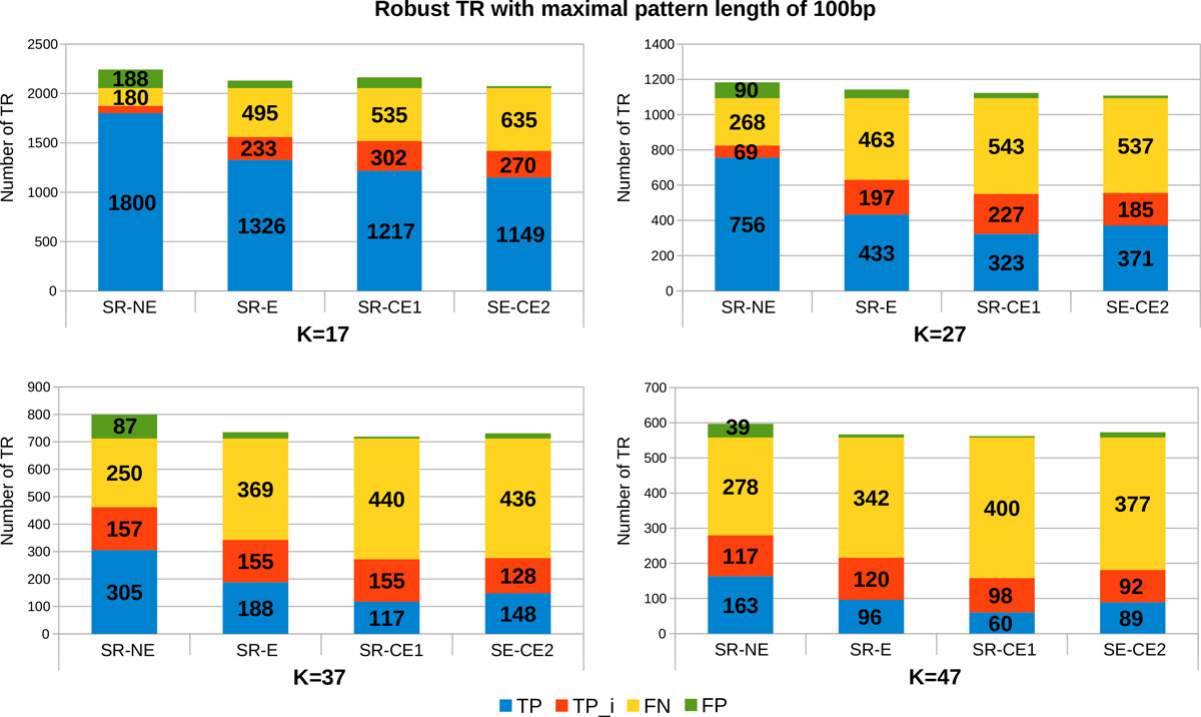
**Number of robust TR with maximal pattern length of 100 bp detected from the first chromosome of *C. elegans***. The results are obtained with the set *LR-E *of long reads with a coverage depth of x20.

The results obtained for the robust TR with *|p| ≤ *100 are described in Figure 7. We obtained with mreps on the first chromosome of *C. elegans *2,054 robust TR for *k *= 17, 1,093 robust TR for *k *= 27, 712 robust TR for *k *= 37 and 558 robust TR for *k *= 47. We observe once again that the quality of results increases when we decrease the value of *k*. The main reason is the one mentioned in the previous paragraph: the fragmentation of the reads creates more cycles and implies a better computation of the arc frequency by MixTaR. Moreover, the correction of *SR-E *can decrease the number of FP obtained with more than 90%. Surprisingly, with these set of short reads (*SR-CE1 *et *SR-CE2*) the number of TP can decrease also, with up to 63% of TP. This is probably due to the error correction methods for the short reads, that are too strict with the short reads base quality.

**Figure 8:**
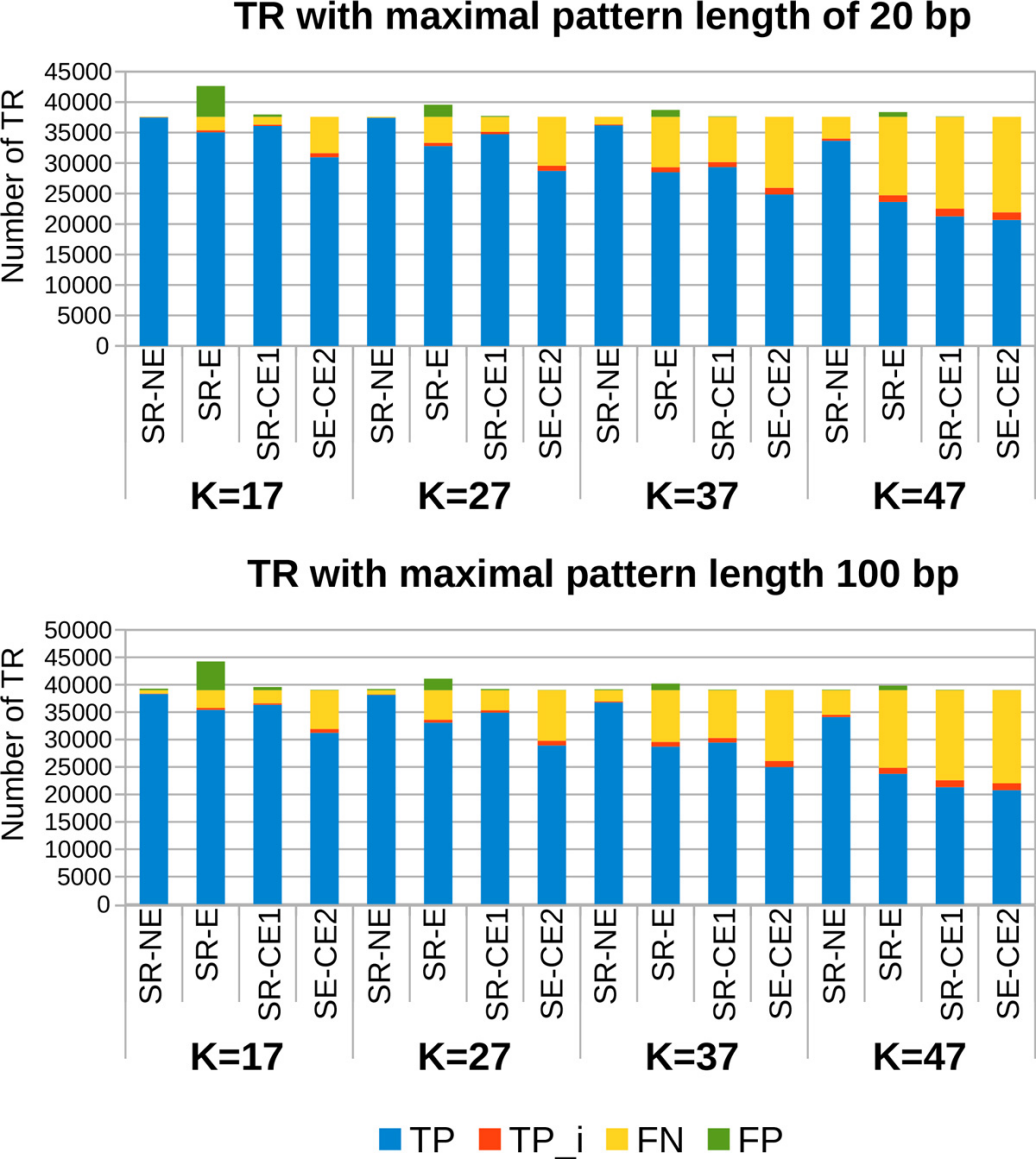
**Number of general TR with maximal pattern length of 20 bp and 100 bp detected from the first chromosome of *C. elegans***. The results are obtained with the set *LR-E *of long reads with a coverage depth of x20.

Figure 8 presents the results obtained on the set of general TR. The efficiency of a small value of *k *is once again proven by the percentage of TP we obtain. We can lose up to 41% of TP when *k *varies from 17 to 47 for a same set of short reads. The values for the precision and the sensitivity obtained for each value of *k *are presented in Table 2. In this table, one can notice that the quality of results obtained for *k *= 17 remains rather stable depending on the percentage of errors from the short reads, the variation of the values for precision being from 0.998 to 0.872. This is due to the fact that the high fragmentation of the reads allows a correct analysis of a significant number of cycles, independently of the error rate of the short reads.

**Table 2:** Precision and sensitivity values for the detection of general TR with maximal pattern length of 100 bp from the first chromosome of *C*.

	K = 17		K = 27		K = 37		K = 47	
	**Prec**.	**Sens**.	**Prec**.	**Sens**.	**Prec**.	**Sens**.	**Prec**.	**Sens**.

**SR-NE**	0.990	**0.984**	0.994	**0.980**	0.996	**0.948**	0.997	**0.884**

**SR-E**	0.872	0.917	0.942	0.862	0.961	0.758	0.968	0.638

**SR-CE1**	0.984	0.939	0.994	0.906	0.997	0.777	0.997	0.579

**SE-CE2**	**0.998**	0.818	**0.999**	0.764	**0.999**	0.669	**0.999**	0.565

*elegans*. The results are obtained with the set LR-E x20 of long reads.

On Figure 8 we can also observe the effect of error-correction procedures on short reads. The number of FP can decrease by more than 90% only by trimming the reads and even with 100% with the error correction method proposed by ALLPATHS. But once again, the number of lost TP can be significant. By correcting the low quality parts of the short reads, the error correcting procedures can remove parts of correct TR. The arc frequency variance is then increased in the cycles formed by these TR, implying supplementary difficulties for MixTaR to correctly identify them.

*The pattern length range of detected TR*. In the results returned by mreps for 2 *≤ |p| ≤ *100, we observe that about 96% of the general TR of the first chromosome of *C. elegans *have 2 *≤ |p| ≤ *20. But the interval 21 *≤ |p| ≤ *100 is also very well covered, since the chromosome has at least one TR for most of the pattern lengths.

**Figure 9:**
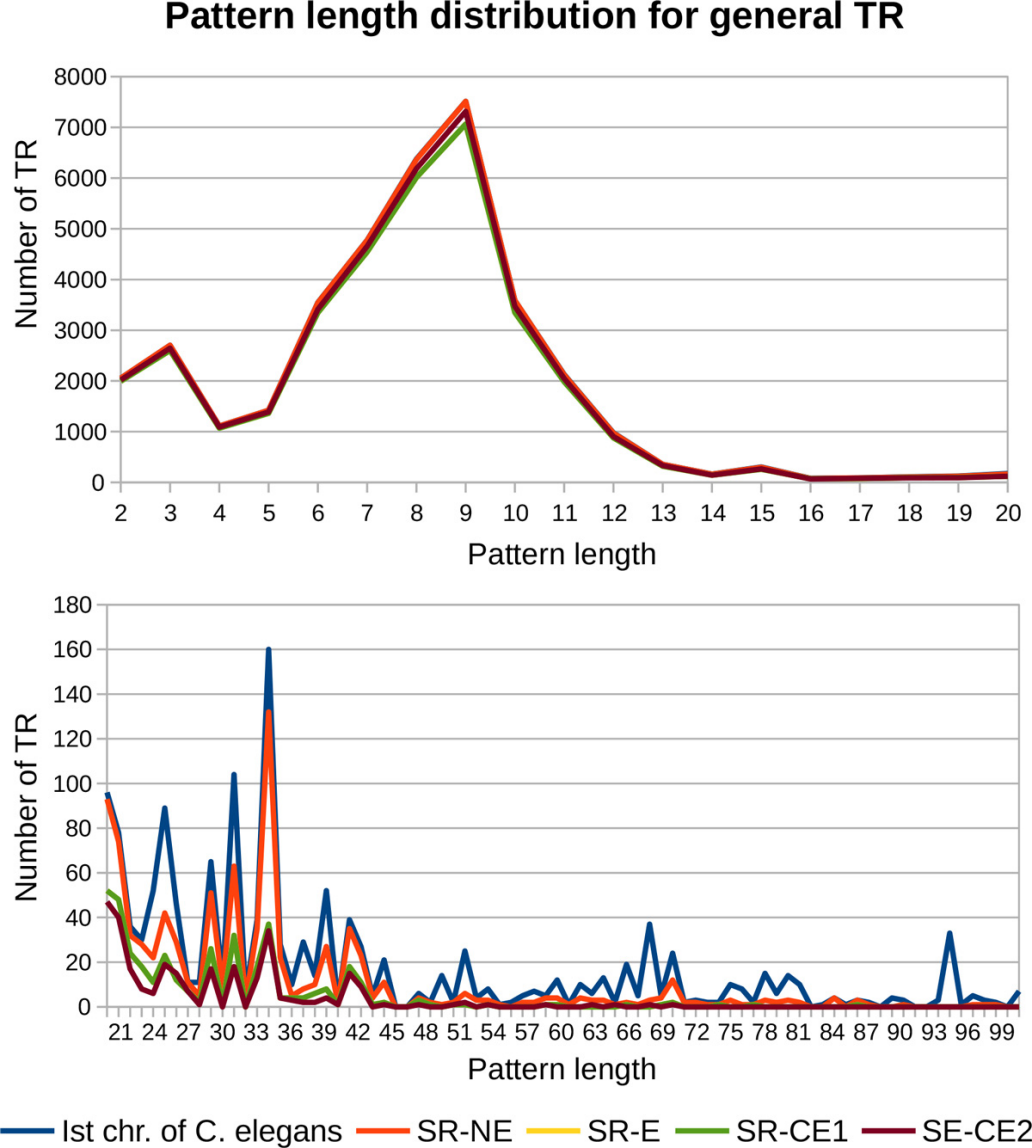
**Pattern length distribution for general TR from the first chromosome of *C. elegans***. The results obtained with MixTaR with the four different sets of short reads, the set *LR-E *x20 of long reads and k = 17 are compared to the ones from the first chromosome of *C. elegans*.

Figure 9 describes the distribution of general TR depending on the pattern length for these two intervals. Along with the TR from the chromosome, we present the results obtained with the four different sets of short reads and for the value of *k *= 17, for which we obtained the highest number of TR. We can notice that on the interval 2 *≤ |p| ≤ *20, the difference on the quality of results obtained with the four different sets of short reads is not very significant. This is due to the fact that MixTaR detects all the cycles of maximal length of 20 vertices for every run and that the fragmentation of the short reads for *k *= 17 allows a correct analysis of them independently of the error rate. On the interval 21 *≤ |p| ≤ *100 though, the sets of erroneous reads allow the detection of less TR than with the set of *SR-NE*. Moreover, the error correction method of ALLPATHS eliminates the possibility of detecting TR with *|p| ≥ *71, while with the sets *SR-E *and *SR-CE1 *we detect TR with *|p| *up to 89 bp and with *SR-NE *even up to 100 bp. This limitation is due to the shorter length of the reads in *SR-CE2 *compared to the three others. Thus, by cutting the short reads, the sequences of TR with long patterns are collapsed and can not form cycles in the de Bruijn graph.

### Real data sets

*Legionella pneumophila *is an intracellular parasite found in human monocytes and responsible of a severe pneumonia known as Legionnaires' disease. Several studies such as [45,46] are focused on the biological role of TR from the genome of *L. pneumophila *and the *Philadelphia *strain.

For our experiments on the strain *Philadelphia*, we used a set of paired short reads obtained with Illumina technology with a coverage depth of approximately 190x [SRA:SRX258262]. In the following, we refer to this set of reads with *SR-RP *for Short Reads Real for *Philadelphia*. Since no long reads sets are available for this strain, we simulated two sets of long reads with PBSIM with a coverage depth of 20x and 100x (*LR-EP *x20 and *LR-EP *x100 for Long Reads with Errors for *Philadelphia*). Two other sets of long reads were obtained by correcting *LR-EP *x20 and *LR-EP *x100 with LoRDEC using the set of real short reads and *k *= 19. These sets are denoted *LR-CEP *x20 and *LR-CEP *x100 for Long Reads with Corrected Errors for *Philadelphia*. The genome has a length of approximately 3.4 Mb [GenBank:GCA 000008485.1].

In order to test MixTaR with both long and short read real data sets, we also used for our experiments a second strain of *L. pneumophila*, the *130b *strain. For our experiments, we used two sets of reads downloaded from the Sequence Read Archive at the NCBI: one set of paired short reads obtained with Illumina sequencing with a coverage depth of approximately 120x [SRA:ERX313832] and a set of long reads obtained with PacBio sequencing [SRA:ERX620205]. In the following, we refer to these two sets of reads with *SR-R130b *(for Short Reads Real for *130b*) and *LR-R130b *(for Long Reads Real for *130b*). A second set of long reads (*LR-RC130b*, for Long Reads Real Corrected for *130b*) was obtained by correcting the set *LR-R130b *with LoRDEC using the set *SR-R130b *and *k *= 19. The draft genome is constituted of 159 contigs [GenBank:GCA 000211115.2].

For the experiments on both strains of *L. pneumophila *that we present, we used the following values for the algorithm parameters. Due to the the short reads length of 100 bp we tested all the odd values for *k ∈ *[17, 47]. In the following paragraphs we present the results obtained for *k *= 17, the value for which we obtained the best results. Since the size of the genome is significantly smaller than the size of the first chromosome of *C. elegans*, the sizes of the set of *k*-mers and of the de Bruijn graph are also smaller. Therefore we included in our cycle search all *k*-mers with at least *σ *= 30 frequency. For the cycle search we set *η *= 10, 000 arcs for cycles of maximal length Λ*max *= 100 and then *λmax *= 30. For the minimum alignment score *τ *we used the value *τ *= 10 for the long reads non-corrected and *τ *= 20 for the corrected ones.

**Figure 10:**
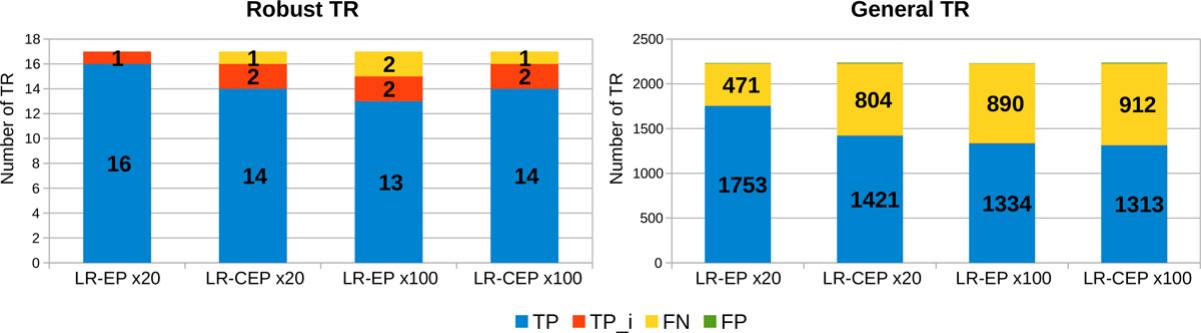
**Number of robust TR and general TR detected from the genome of *L. pneumophila *(*Philadelphia *strain)**. The results are obtained with the real set of short reads *SR-RP *and k = 17.

*Results obtained on the Philadelphia strain of L. pneumophila *On the *Philadelphia *strain mreps obtained 2,227 TR with 2 *≤ |p| ≤ *100. Among these TR, only 17 are robust TR for *k *= 17. MixTaR detects, depending on the set of LR we use, at least 13 of them completely, as shown in Figure 10. Note that there are no FP. The TR detected by our algorithm are either complete (*T P) *or with a lower number of copies but correctly located on the *Philadelphia *strain (*T Pi*). This is explained by the selection of significant short reads in the local assemblies. The greedy assemblies can introduce FP when two overlapping reads are not located in the same region of the genome. As mentioned before, this case appears mostly because of repeats. Since the *Philadelphia *strain contains a low number of repeats and since we limit the number of short reads used in the assemblies, the number of FP is significantly low. This behaviour is also noticed for the general TR, with less than 0.75% of FP on the sets of general TR detected by MixTaR. However, the percentage of general TR detected by our algorithm is lower that the one obtained for *C. elegans*. This is explained by the fact that the general TR on the *Philadelphia *strain are not always located in the flanking regions of robust TR or having the same pattern with a robust TR. We can miss up to 30% of the general TR from the *Philadelphia *strain. Unlike for the first chromosome of *C. elegans*, the difference between the results quality obtained with the non-corrected and corrected long read sets is now more significant. The quality difference is also underlined by the values for sensitivity from Table 3. The main reason is the use of real short read set for the error correction procedure of the long reads. LoRDEC is not able to detect all the errors from the TR in the long reads.

**Table 3:** Precision and sensitivity values for the detection of robust TR and of general TR from the genome of *L*.

	Robust TR		General TR	
	**Precision**	**Sensitivity**	**Precision**	**Sensitivity**

**LR-EP x20**	**1**	**1**	**0.997**	**0.789**

**LR-CEP x20**	**1**	0.941	0.993	0.639

**LR-EP x100**	**1**	0.882	**0.997**	0.600

**LR-CEP x100**	**1**	0.941	0.992	0.590

*pneumophila *(*Philadelphia *strain).

**Figure 11:**
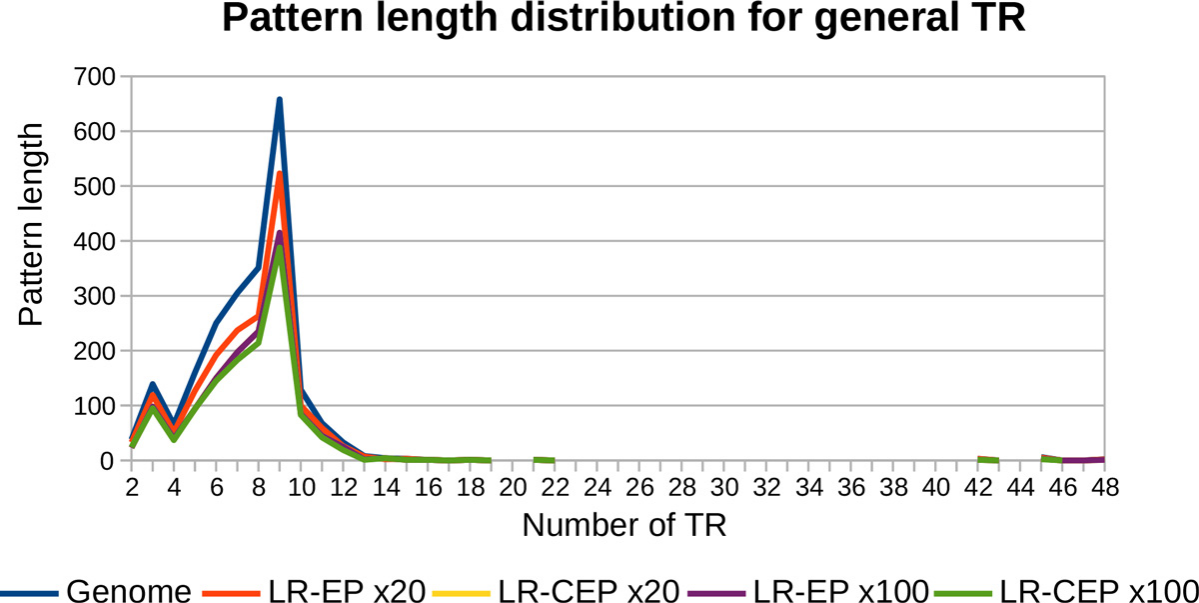
**Pattern length distribution for general TR detected from the genome of *L. pneumophila *(*Philadelphia *strain)**. The results obtained with MixTaR with the four different sets of long reads, the real set *SR-RP *of short reads and k = 17 are compared to the ones from the *Philadelphia *strain.

The TR of the *Philadelphia *strain have patterns with lengths ranging from 2 to 48, as presented in Figure 11. Due to the length of the analysed cycles, our algorithm is able to cover the entire pattern length interval.

Several TR from the *Philadelphia *strain of *L. pneumophila *were studied for their biological significance [45,46]. We present in Table 4 the results obtained by MixTaR for all the TR with 2 *≤ |p| ≤ *100 presented in [45,46]. The set of TR from the *Philadelphia *strain used in the results presented before in this paragraph was obtained by mreps with the highest value for the alignment score between the copies of an ATR. Papers [45,46] do not specify the exact sequence of the analysed TR, but only the approximate location on the strain (the gene), the pattern length and the number of copies. Thus, in order to retrieve their sequence, we run mreps on the specified regions (genes) of the *Philadelphia *strain and we decreased the alignment score parameter until finding a TR corresponding to the pattern length and the number of copies given in [45,46]. The name of the TR and of the genes presented in Table 4 are the ones mentioned in [45,46]. We then searched their sequences on the results obtained by MixTaR, and, as presented in Table 4 most of the TR are completely detected by our algorithm. An additional table shows the exact sequences and positions returned by mreps for the TR presented in Table 4 [see Additional file 1].

**Table 4:** Biologically significant TR detected by MixTaR from the genome *L*.

Name	Gene	Pattern length	Copy number	LR-EP x20	LR-CEP x20	LR-EP x100	LR-CEP x100
	LPG0451	30 bp	5.9	Complete	-	-	-
	LPG0688	9 bp	6	Complete	Complete	Complete	Complete
	LPG1038	12 bp	4.17	Complete	Complete	Complete	Complete
Lpms35	LPG1299	18 bp	3	Complete	-	-	-
	LPG1555	21 bp	2	Complete	Complete	Complete	Complete
	LPG1602	90 bp	9.2	Complete	Complete	Complete	Complete
	LPG1948	90 bp	7.08	Complete	-	Complete	-
	LPG1958	87 bp	13.59	Complete	-	Complete	-
	LPG2392	87 bp	6.49	-	-	-	-
	LPG2559	12 bp	4.08	Complete	Complete	Complete	Complete
Lpms31	LPG2644	45 bp	19.44	Incomplete	Incomplete	Incomplete	Incomplete
				(11 copies)	(11 copies)	(11 copies)	(11 copies)
Lpms3	LPG2793	96 bp	7.58	Complete	-	-	-
Lpms01	LPG2854	45 bp	7.64	Incomplete	-	-	-
				(6 copies)			
Lpms_1_3	LPG1488	24 bp	9.75	Incomplete	Incomplete	Incomplete	Incomplete
				(6 copies)	(6 copies)	(6 copies)	(6 copies)
Lpms_1_7	LPG0854	89 bp	2.28	-	Complete	-	Complete
Lpms_1_9	Intergenic	21 bp	4.05	-	Complete	-	Complete

*pneumophila *(*Philadelphia *strain) and presented in [45,46].

**Name Gene Pattern length Copy number LR-EP x20 LR-CEP x20 LR-EP x100 LR-CEP x100**

**Figure 12:**
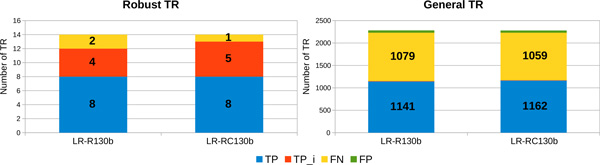
**Number of robust TR and general TR detected from the genome of *L. pneumophila *(*130b *strain)**. Results obtained with the real set *SR-R130b *of short reads and k = 17.

*Results obtained on the 130b strain of L. pneumophila*. After running mreps on the contigs of the *130b *strain for TR with 2 *≤ |p| ≤ *100, we obtained 2,230 TR. Only 14 of them are robust TR for *k *= 17. As on the *Philadelphia *strain, MixTaR obtains good results, as shown in Figure 12. Once again, our algorithm outputs no FP and the TR it detects are either complete (*T P) *or with a lower number of copies but correctly located on the *130b *strain (*T Pi*). In spite of the small number of TR that can form a cycle in the de Bruijn graph for *k *= 17, MixTaR is able to detect more than 51% of all the TR detected by mreps on the genome (Figure 12). As before, the TR that are missing from our output have either patterns very different from the ones of the robust TR, or are located at a significant distance from these. Also, the lower number of robust TR of the *130b *strain compared to the *Philadelphia *strain decreases the chances for MixTaR to detect general TR.

The results obtained by our algorithm contain a low percentage of FP. With the set *LR-R130b *only 8.8% of the TR detected are FP, the value decreasing to 8.6% for the set *LR-RC130b*. The precision and sensitivity values for both sets of TR are presented in Table 5. Contrary to results obtained with the long reads on the *Philadelphia *strain, MixTaR showed slightly better results on the set of corrected long reads than on the one non-corrected. This is probably caused by a higher error rate of the real long reads than of the simulated ones.

**Table 5:** Precision and sensitivity values for the detection of robust TR and of general TR from the genome of *L*.

	Robust TR		General TR	
	**Precision**	**Sensitivity**	**Precision**	**Sensitivity**

**LR-R130b**	**1**	0.857	0.957	0.516

**LR-RC130b**	**1**	**0.929**	**0.959**	**0.525**

*pneumophila *(*130b *strain).

**Figure 13:**
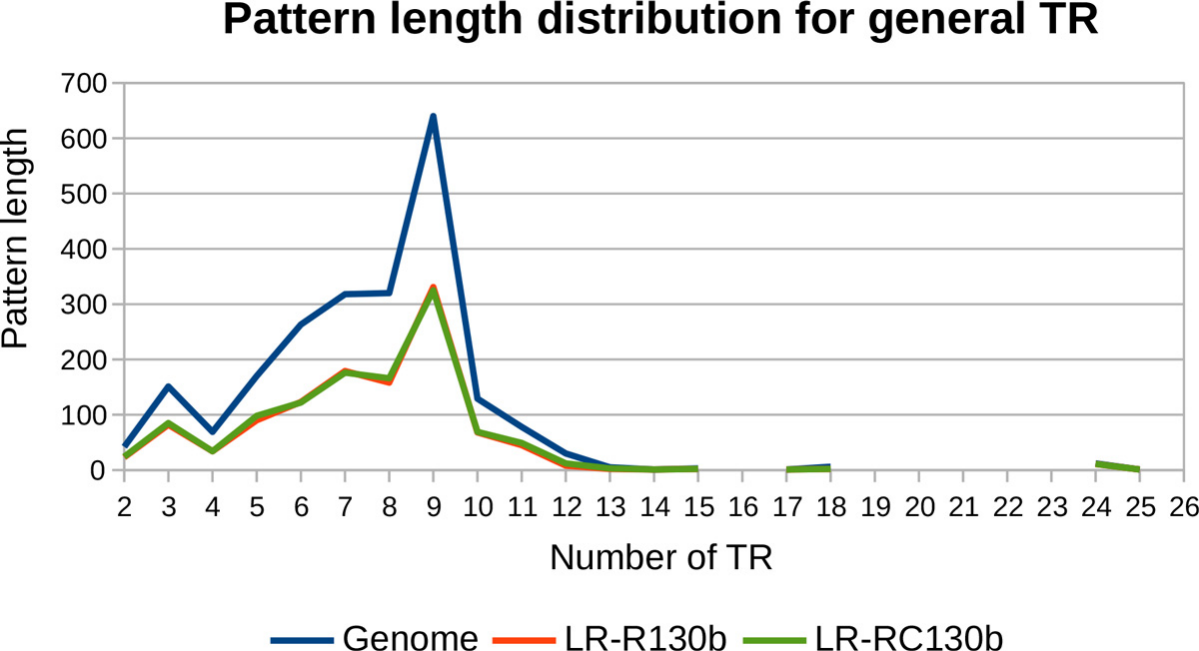
**Pattern length distribution for general TR detected from the genome of *L. pneumophila *(*130b *strain)**. The results obtained with MixTaR with the two different sets of long reads, the real set *SR-R130b *of short reads and k = 17 are compared to the ones from the *130b *strain.

The TR of *L. pneumophila *have patterns with length ranging from 2 to 25, as presented in Figure 13. Again, due to the length of the analysed cycles, the results obtained with both sets of long reads cover the entire pattern length interval.

## Conclusion

This paper presents a new algorithm, MixTaR, that represents an efficient solution to the problem of de novo detection of TR. The method focuses only on the parts of the genome where potential TR can be located, and does not compute global assemblies. By mixing the quality of short reads with the length of long reads, we introduced a robust approach. We obtained high quality results on complex organisms, and using sets of reads with different error rates. Keeping low false positive rates, our method detects accurate TR with pattern lengths varying within a significant interval.

MixTaR identifies a significant number of TR on both real and simulated read sets. However, the complexity of the target DNA fragment influences the amount of general TR detected. As mentioned before, the initial target imposed by our approach is the set of robust TR. By assembling the short reads spanning the robust TR patterns, we are able to identify a significant amount of non-robust TR. This is due either to the fact that they are located in the flanking regions of the robust TR, or to the similarity between the patterns of non-robust and robust TR. As a consequence, the number of detected TR increases with the number of robust TR presented in the target DNA fragment. Thus, the performances of MixTaR increase with the complexity of the target DNA fragment.

Future improvements and extensions are planned to be included in our algorithm and in our study. Each tool that detects TR in a reference DNA sequence has its own definition of ATR and the sets of detected TR are different [8]. In this paper, we used *mreps *[10] for our analysis, but the study can be extended to other TR search tools, for instance, *Tandem repeats finder *[11].

Also, the results are presented in this paper from the point of view of the TR sequences. The algorithm and also the TR quality study can be extended to include the flanking regions of the TR. Thus, instead of only comparing TR sequences between our results and the target DNA sequence, we can also provide more information concerning the location of each TR.

## List of abbreviations

SGS: Second Generation Sequencing; TGS: Third Generation Sequencing; TR: Tandem repeat; ETR: Exact tandem repeat; ATR: Approximate tandem repeat; SR: set of Short Reads obtained with a SGS technology; LR: set of Long Reads obtained with Pacific Bioscience sequencing technology.

## Competing interests

The authors declare that they have no competing interests.

## Authors' contributions

The algorithm and the experiments described in this paper emerged from discussions between all the authors. AR implemented the algorithm, carried out the experiments and wrote the manuscript. GF, GJ and IR edited the manuscript. All authors read and approved the final manuscript.

## Supplementary Material

Additional file 1 Sample additional file title. Positions and sequences for the biologically significant TR detected by MixTaR on the *Philadelphia *strain of *L. pneumophila *and presented in [45,46]. Click here for file

## Declarations

Publication costs for this article were funded by bioinformatics theme from LINA UMR CNRS 6241, University of Nantes.

This article has been published as part of BMC *Medical Genomics *Volume 8 Supplement 3, 2015: Selected articles from the IEE International Conference on Bioinformatics and Biomedicine (BIBM 2014): Medical Genomics. The full contents of the supplement are available online at http://www.biomedcentral.com/bmcmedgenomics/supplements/8/S3.
